# Supplementary material for: Evolutionary signatures of human cancers revealed via genomic analysis of over 35,000 patients
Source: Nat Commun. 2023 Sep 25;14:5982. doi: 10.1038/s41467-023-41670-3 (PMC10519956; doi:10.1038/s41467-023-41670-3)
Supplement: Supplementary file 3 — Description of Additional Supplementary Files [file 41467_2023_41670_MOESM3_ESM.pdf]

File Name: Supplementary Data 1
Description: Evolutionary steps inferred by ASCETIC in Acute Myeloid Leukemia (Tapestri).

File Name: Supplementary Data 2
Description: The number at risk for all the survival Kaplan-Meier curves presented in the study.

File Name: Supplementary Data 3
Description: Evolutionary steps inferred by ASCETIC in Non-small cell lung cancer (TRACERx).

File Name: Supplementary Data 4
Description: Evolutionary steps inferred by ASCETIC in the Pan-Cancer Atlas dataset.

File Name: Supplementary Data 5
Description: Evolutionary steps inferred by ASCETIC in the MSK-MET dataset.

File Name: Supplementary Data 6
Description: Shared evolutionary steps at the pan-cancer level in both the Pan-Cancer Atlas dataset and MSK-MET dataset.
